# Reduction of pedestrian death rates: a missed global target

**DOI:** 10.1186/s13017-020-00315-2

**Published:** 2020-05-19

**Authors:** Yasin J. Yasin, Michal Grivna, Fikri M. Abu-Zidan

**Affiliations:** 1 Institute of Public Health, College of Medicine and Health Sciences, UAE University, Al-Ain, United Arab Emirates; 2 Department of Environmental Health and Behavioral Sciences, School of Public health, College of Health Sciences, Mekelle University, Mekelle, Ethiopia; 3 Department of Surgery, College of Medicine and Health Sciences, UAE University, Al-Ain, United Arab Emirates

**Keywords:** Global, Pedestrian, Death, Road traffic collision, Road safety

## Abstract

**Background:**

The UN Decade of Action for Road Safety aimed to reduce road traffic deaths by half by year 2020. We aimed to study risk factors affecting global pedestrian death rates overtime, and whether the defined target of its reduction by WHO has been achieved.

**Methods:**

The studied variables were retrieved from the WHO Global Status Reports on Road Safety published over 2010–2018. These covered years 2007–2016 and included the estimated road traffic death rates per 100,000 population, policies to promote walking and cycling, enforcement levels of national speed limits, the gross national income per capita and the vehicle/person ratio in each country. A mixed linear model was performed to define the factors affecting the change of pedestrian death rates overtime.

**Results:**

Global pedestrian mortality decreased by 28% over 10 years. This was significant between years 2007 and 2010 (*p* = 0.034), between years 2013 and 2016 (*p* = 0.002) but not between 2010 and 2013 (*p* = 0.06). Factors that reduced pedestrian death rates included time (*p* < 0.0001), GNI (*p* < 0.0001), and vehicle/person ratio (*p* < 0.0001). There was a significant drop overtime in both the middle-income, and high-income countries (*p* < 0.0001, Friedman test), but not in the low-income countries (*p* = 0.35, Friedman test).

**Conclusions:**

Global pedestrian mortality has dropped by 28% over a recent decade, which is less than the 50% targeted reduction. This was mainly driven by improved GNI and using more vehicles. The economical gap between poor and rich countries has a major impact on pedestrian death rates.

## Introduction

Road traffic collision (RTC) is a major global health problem. During 2016, it was ranked as the sixth cause of premature death and as the eighth cause of death of all ages [1, 2] with 1.35 million annual deaths worldwide [2]. However, if proper actions were not taken, it will be the fifth leading cause of death by the year 2030 [3, 4].

Walking is a common cheap transportation method in developing countries having health benefits [5]. Nevertheless, pedestrians are the most vulnerable road users [6, 7]. The increased number of vehicles combined with low pedestrian safety increases the risk of pedestrian injuries. Pedestrian death rates are high and account for more than 20% of all road traffic deaths [2, 8].

The United Nations General Assembly approved the target to reduce road traffic deaths by half, saving 5 million lives by year 2020, as part of its Decade of Action for Road Safety 2011-2020 [4, 9]. Achieving such target needs continuous monitoring of global progress to evaluate the plan and refine it [10]. The World Health Organization (WHO) Global Status Reports on Road Safety serve such important role [2, 3, 11, 12]. We have previously reported the global burden of pedestrian injuries up to 2010 and factors affecting them [8]. We think that it is time now to see the recent progress over time. Accordingly, we aim to study risk factors affecting global pedestrian death rates over a recent decade to find whether the defined target of its reduction by WHO has been achieved.

## Methods

### Ethical consideration

Data used are publicly available published data from the WHO and do not need approval from the human research ethics committee.

### Data collection

Data used in this study were retrieved from the available WHO Global Status Reports on Road Safety for years 2007, 2010, 2013, and 2016 [2, 3, 11, 12]. These reports are regularly published every 2–3years. The last published report was in 2018 [2], which included data for year 2016. The 2007, 2010, 2013, and 2016 reports had data on 178, 181, 180, and 175 countries, respectively. Complete data on pedestrian mortality was available for 136 (76%), 134 (74%), 140 (78%), and 129 (74%) countries, respectively. The area of the countries was retrieved using the website of infoplease.com [13].

### Studied variables

Studied variables included the country population, the reported number of road traffic deaths, the estimated road traffic death rate per 100,000 population, the percentage of pedestrian deaths out of all road traffic collision deaths, the presence of policies to promote walking and cycling, the effectiveness of overall enforcement levels of national speed limits, the Gross National Income (GNI) per capita in US dollars, and the number of registered vehicles in each country.

Information on the presence of policies to promote walking and cycling was ranked from 0 to 2 where no = 0 for countries have no policy, subnational = 1 for countries having partial policy, and yes = 2 for countries have a clear policy. The effectiveness of enforcement levels of speed limits was scored on a scale of 0 to 10, where 0 is “not effective” and 10 is “highly effective” based on professional opinion of government respondents [2, 3, 11, 12]. Data were entered into excel program and rechecked for accuracy before data analysis.

### Calculations

Population density (number of people/mile square) was calculated by dividing country population by country area. Pedestrian death rates were calculated by multiplying the estimated road traffic death rates per 100,000 population by percentage of pedestrians deaths. Vehicle per person ratio was calculated by dividing the number of registered vehicles by country population.

### Statistical analysis

A mixed linear model (MLM) was performed to define the factors affecting the change of pedestrian death rates over time. This model analyses the data of each country separately taking into consideration both the slope and intercept of each linear line of a country. We have used the strict unstructured model, which assumes that both the variance at each studied year and the correlation (covariance) between studied independent factors are different. We have taken that decision after graph exploration of the data and noticing that the change of pedestrian death rates differed between low, middle-, and high-income countries.

MLM needs to have a normal distribution of the outcome dependent variable. The independent covariates can be continuous, ordinal, or binomial and do not need to have a normal distribution. The MLM analyses would address the nonlinear relationship between different factors. The logarithmic transformation of pedestrian death rate had the best normal distribution and was used for the analysis. The distribution of the log transformation was also normal within each year.

Log transformation of pedestrian death rate was the dependent variable. Its change was studied overtime (factor = year) while independent variables were entered as covariates (continuous variables included GNI per capita, density of population, and vehicle per person ratio; while ordinal data included enforcement of speed legislation (0–10) and promoting alternative transport (0–2)). The MLM included type III sum of squares error because the data were unbalanced. Interactions between GNI and vehicle/person ratio, speed legislation, and promoting alternative transport were tested in different models. The interactions were non-significant and were excluded from the final main effects model.

After achieving the results of the MLM model, univariate post hoc analyses were performed to explain the findings of the MLM model. To do that, Spearman rank correlation test was used to study the correlation between different variables. Friedman test was used to compare more than two dependent groups having continuous data. Wilcoxon signed-rank test was used to compare two dependent groups having continuous data. Data were analyzed with the IBM SPSS Statistics version 26 (SPSS Inc, Chicago, IL, USA). A *p* value of less than 0.05 was accepted as significant.

## Results

**Fig. 1:**
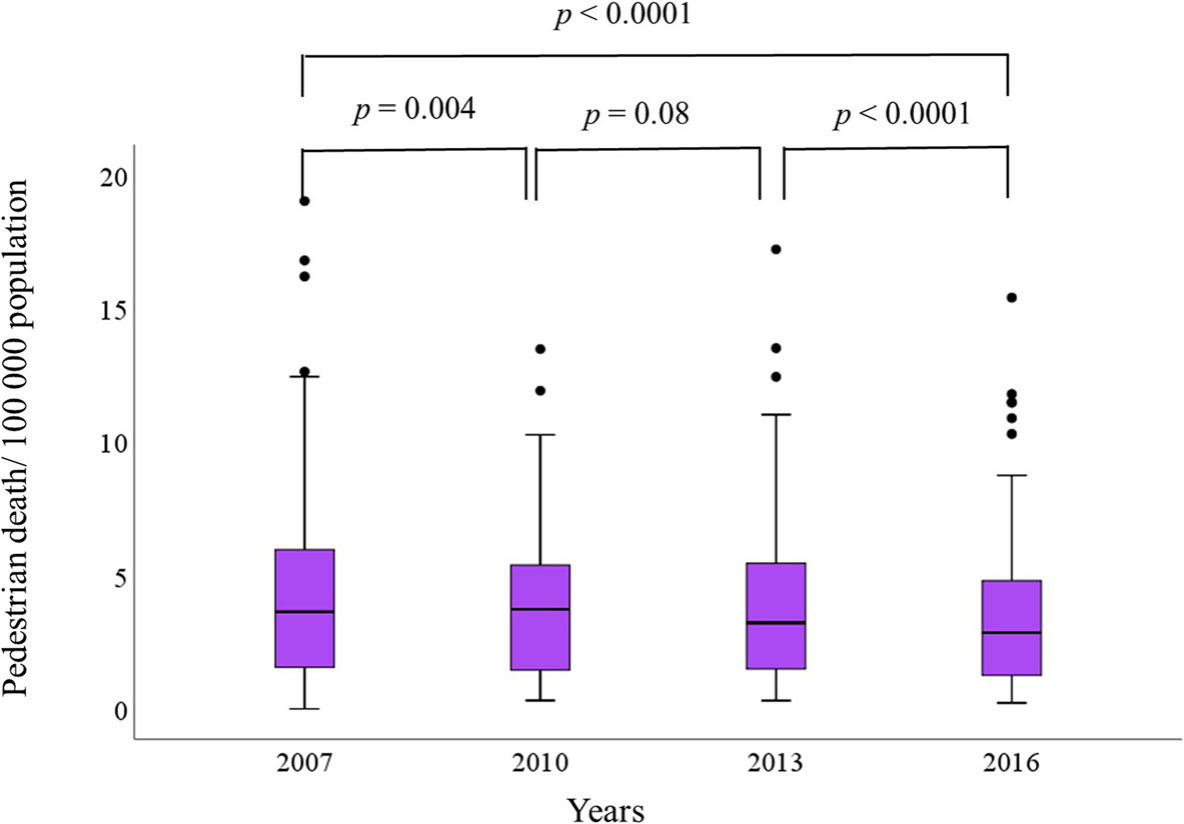
Box-and-whiskers plot of global pedestrian death rate/100,000 population of years 2007–2016. The box resembles the 25th percentile and the 75th percentile Interquartile Range (IQR). While the line within the box resembles the median. Black circles represent the outliers. *p* value = Friedman test for comparison of more than two dependent groups and Wilcoxon signed rank test for comparison of two dependent groups

Table 1 shows the results of the mixed linear model. The model has shown that factors that affected the log transformation of pedestrian death rates included time (*p* < 0.0001), GNI (*p* < 0.0001), and vehicle/person ratio (*p* < 0.0001). There was a significant drop of mortality between years 2007 and 2010 (*p* = 0.034), between years 2013 and 2016 (*p* = 0.002) but not between 2010 and 2013 (*p* = 0.06). We confirmed this finding using the post hoc analysis (Fig. 1). There was statistical significant drop of pedestrian death rate overtime (*p* < 0.0001, Friedman test). The drop was significant between years 2007 and 2010 (median (IQ range): 4.21 (1.66–7.01) compared with 3.95 (1.48–6.23) per 100,000 population (*p* = 0.004, Wilcoxon signed-ranks test), between years 2013 and 2016 (median (IQ range): 3.73 (1.59–6.2) compared with 3.03 (1.39–5.35) per 100,000 population (*p* < 0.0001 = Wilcoxon signed ranks test) but not between years 2010 and 2013 (median (IQ range), 3.95 (1.48–6.23) compared with 3.73 (1.59–6.2) per 100,000 population (*p* = 0.08, Wilcoxon signed-ranks test). The percentage of drop of mortality was 6.2% between 2007 and 2010, 5.6% between 2010 and 2013, and 18.8% between 2013 and 2016. The overall percentage drop of mortality rate was 28%.

**Table 1:** Linear mixed effect model of factors affecting log transformation of pedestrian death rate globally over a decade 2007–2016

Variable	Estimate	SE	*t* value	*p* value	95% CI lower limit	95% CI upper limit
Year 2007	0.069	0.032	2.155	0.034	0.006	0.133
Year 2010	0.046	0.024	1.876	0.064	− 0.003	0.094
Year 2013	0.075	0.024	3.190	0.002	0.029	0.122
Gross national income/capita	− 7.893^-6^	1.428^-6^	− 5.526	< 0.0001	− 1.071^−5^	− 5.078^−6^
Enforcement of speed legislation	− 0.007	0.006	− 1.163	0.246	− 0.019	0.005
Promoting alternative transport	− 0.021	0.014	− 1.525	0.128	− 0.048	0.006
Density of population	− 2.143^−5^	1.231^−5^	− 1.742	0.084	− 4.578^−5^	2.914^−6^
Vehicle/person ratio	− 0.441	0.091	− 4.855	< 0.0001	− 0.619	− 0.262
Intercept	0.781	0.049	15.985	< 0.0001	0.685	0.879

*SE* standard error, *CI* confidence interval

**Fig. 2:**
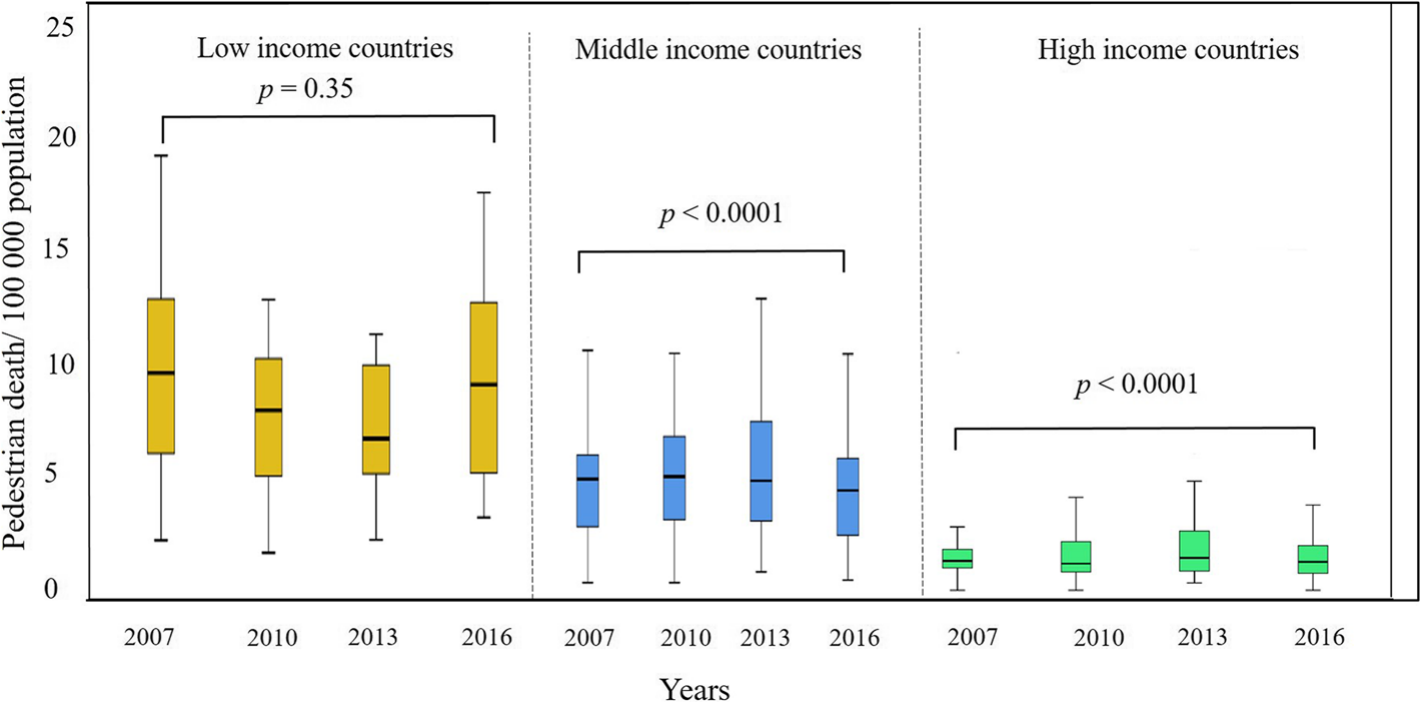
Box-and-whiskers plot of global pedestrian death rate/100,000 population of years 2007–2016 by level of income of countries. The box resembles the 25th percentile and the 75th percentile Interquartile Range (IQR). While the line within the box resembles the median. *p* value = Friedman test for comparison of more than two dependent groups

**Fig. 3:**
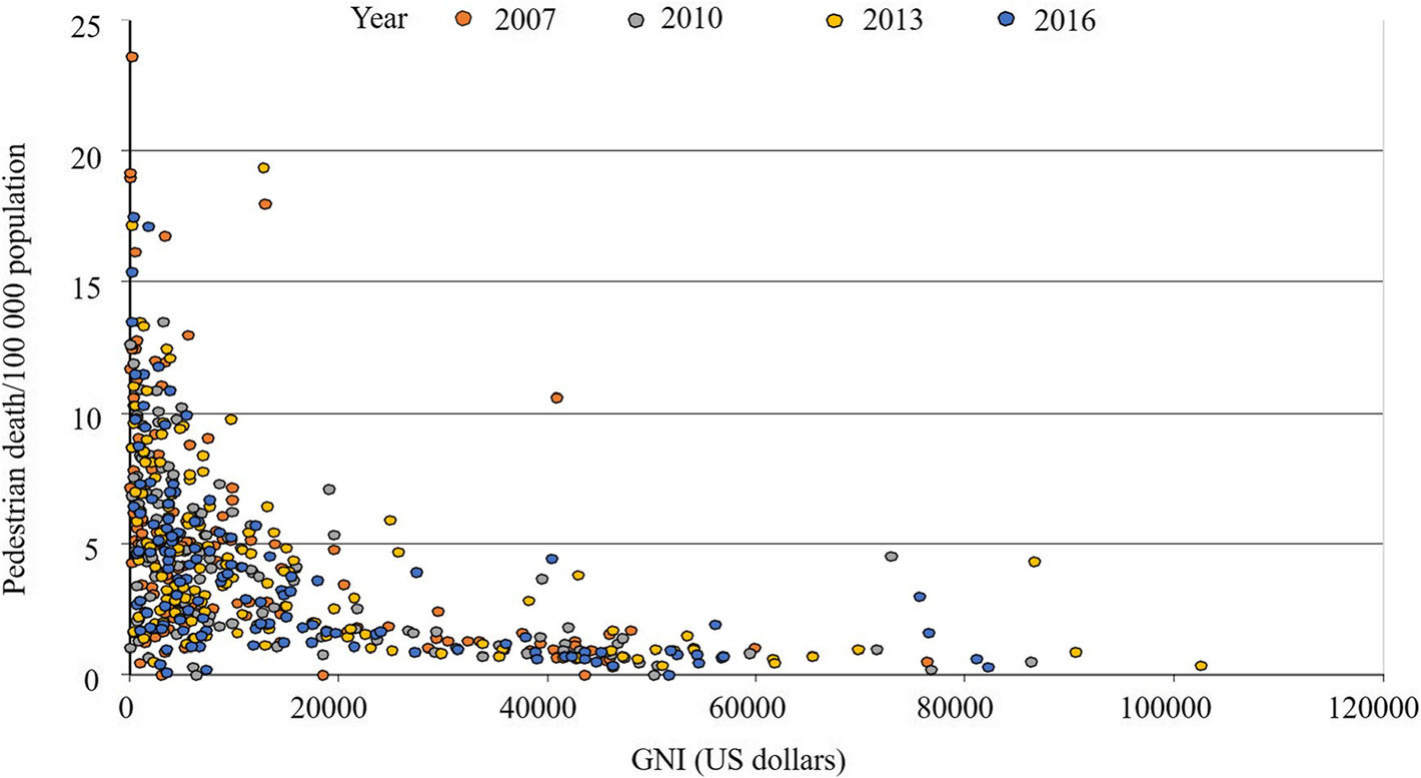
The correlation (Scatter plot) between pedestrian death rates/100,000 population and Gross National Income (GNI) by US dollars per capita during the period 2007–2016

**Fig. 4:**
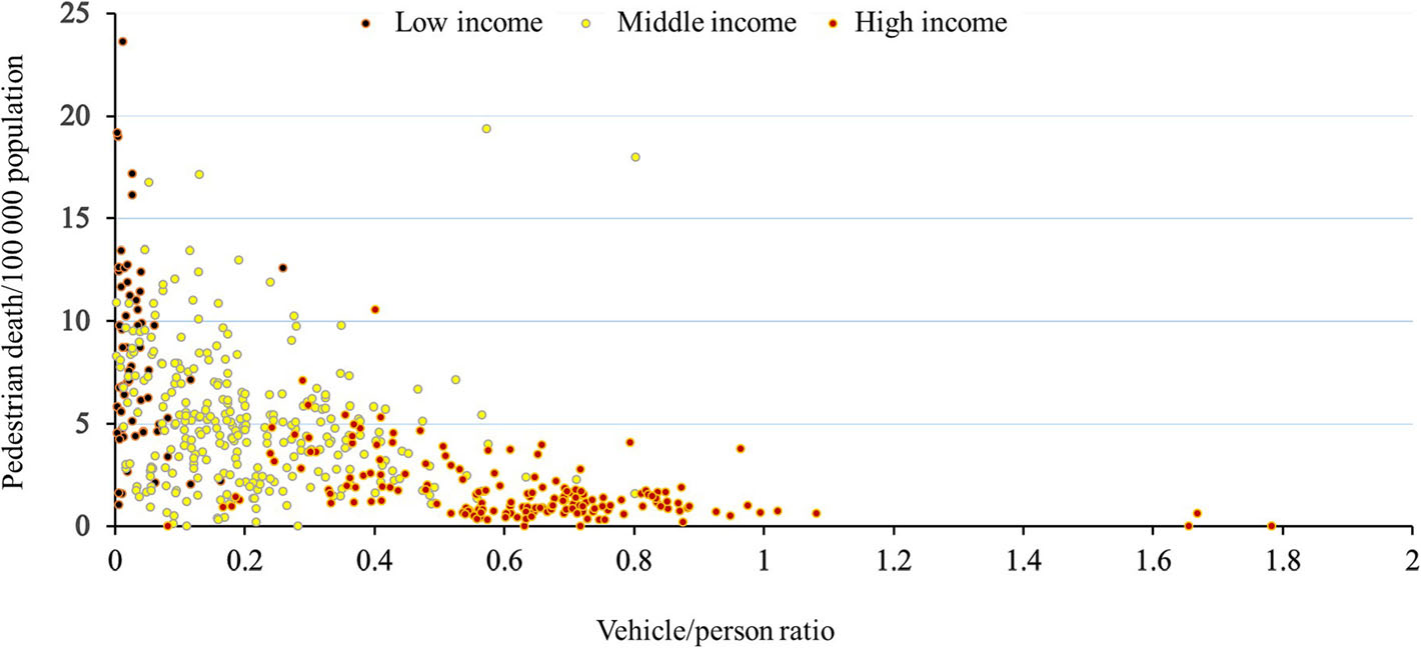
The correlation (scatter plot) between pedestrian death rates/100,000 population and vehicle/person ratio during the period 2007–2016 by income level of the countries

When compared by the income level (Fig. 2), there was a significant drop overtime in both the middle-income (*n* = 53) and high-income countries (*n* = 31) (*p* < 0.0001, Friedman test), but not in the low-income country (*n* = 14) (*p* = 0.35, Friedman test). There was a highly significant correlation between the pedestrian death rate and the GNI (Spearman rank correlation, rho = − 0.65, *p* < 0.0001) (Fig. 3), between the pedestrian death rate and vehicle per person ratio (Spearman rank correlation, rho = − 0.64, *p* < 0.0001) (Fig. 4), and between GNI and vehicle per person ratio (Spearman rank correlation, rho = 0.88, *p* < 0.0001). These findings have been consistent through all studied years (Table 2).

**Table 2:** Spearman rank correlations between the significant factors that affected the global pedestrian death rate during the period 2007–2016

Variable	Vehicle/person ratio		GNI per capita	
	rho	*p* value	rho	*p* value
Year 2070				
Pedestrian death rate	− 0.6	*p* < 0001	− 0.63	*p* < 0001
GNI	0.91	*p* < 0001	–	–
Year 2010				
Pedestrian death rate	− 0.67	*p* < 0001	− 0.65	*p* < 0001
GNI	0.87	*p* < 0001	–	–
Year 2013				
Pedestrian death rate	− 0.64	*p* < 0001	− 0.64	*p* < 0001
GNI	0.86	*p* < 0001	–	–
Year 2016				
Pedestrian death rate	− 0.65	*p* < 0001	− 0.68	*p* < 0001
GNI	0.86	*p* < 0001	–	–

*GNI* Gross National Income (US dollars)/capita

## Discussion

Our study has shown that the global pedestrian mortality has dropped by more than 25% over a recent decade. This drop is affected by GNI which reflected by the vehicle per person ratio. However, the drop is not uniform in all countries and it was mainly in middle- and high-income countries compared with low-income countries.

Pedestrian injuries cause 23% of RTC mortality worldwide [2]. However, 45%, of these deaths occur in low-income countries compared with 29% and 18% in middle-and high-income countries [14]. The drop of mortality by 25% over a recent decade indicates partial success of the global plan, which did not reach the defined target of 50%. It is predicted that pedestrian death will increase in low-income countries and decrease in middle- and high-income countries in the coming 10 years, with an overall global decrease of 10% [15].

High GNI was an important factor in reducing pedestrian deaths in this study. This is because the increase in GNI increases the number of motor vehicles [16] and improves the construction of safer roads with signaled pedestrian crosswalks, humps, and road traffic cameras [17–19]. The increased vehicle per person ratio is associated with reduced pedestrian road users and their death rates. GNI is associated with a long-term decrease of road traffic deaths because of economic development despite the initial early increase of deaths [20]. This is attributed to increased number of transport users at the beginning of economic transition [21]. Following this period, investment in the health care system such as pre-hospital transportation, trauma centers, surgical care, and rehabilitation will reduce pedestrian death [20, 22].

Our study is similar to other studies showing that increased GNI reduces pedestrian death rate [8, 23]. Nevertheless, this was not consistent through the whole decade. There was a slowdown of this effect during the period 2010–2013 possibly related to the economical slow down at that period. Furthermore, our previous study showed that speed control and decreased population density significantly reduced pedestrian death rates [8] which was supported by others [24, 25]. This effect has disappeared in the current study. It indicates that these effects have been stabilizsed over time and other important factors were recognized.

### Limitations of the study

We have to acknowledge that our study has certain limitations. *First*, the WHO reports included limited available variables. There are individual important factors missing, such as pedestrian behavior [5, 26], educational level, gender, age, alcohol use [7, 27, 28], use of visibility aids at night [29], and pedestrian friendly vehicles pumpers [30, 31]. Nevertheless, this is a global study on country levels and not individual levels and these factors are difficult to quantify on this level. *Second*, data of the WHO reports takes up to 3 years to publish. The report on the last 3 years has not yet been published and not included in the present study. *Third*, GNI is a single collective factor that reflects other embedded factors such as education, road user behavior, and risk perception of danger [8]. *Finally*, our analysis was based on the WHO reported data on pedestrian mortality and other covariates. These reports depend on data availability and the official reporting of countries, which can be affected by poor health informatics and political interests especially in low-income developing countries [10, 32, 33].

## Conclusions

Global pedestrian mortality has dropped by more than 25% over a recent decade, which did not reach the defined target of 50%. This was mainly driven by improved GNI and using more vehicles. The drop in mortality was clear in middle- and high-income countries compared with low-income countries. The economical gap between poor and rich countries has a major impact on pedestrian death rates.

## Data Availability

Original data are published by the WHO and available on the website (references [2, 3, 11, 12]).

## Abbreviations

GNI: Gross National Income
MLM: Mixed linear model
RTC: Road traffic collision
WHO: World Health Organization
